# scBCN: deep learning-based batch correction network for integration of heterogeneous single-cell data

**DOI:** 10.1093/bib/bbaf503

**Published:** 2025-09-24

**Authors:** Lei Wan, Yang Zhou, Xingzhi Wang, Jing Qi, Shuilin Jin

**Affiliations:** School of Mathematics, Harbin Institute of Technology, No. 92 West Dazhi Street, Harbin, Heilongjiang 150001, China; Zhengzhou Research Institute, Harbin Institute of Technology, No. 26 Longyuan East 7th Street, Zhengzhou, Henan 450000, China; School of Mathematics, Harbin Institute of Technology, No. 92 West Dazhi Street, Harbin, Heilongjiang 150001, China; Zhengzhou Research Institute, Harbin Institute of Technology, No. 26 Longyuan East 7th Street, Zhengzhou, Henan 450000, China; School of Mathematics, Harbin Institute of Technology, No. 92 West Dazhi Street, Harbin, Heilongjiang 150001, China; School of Mathematics, Harbin Institute of Technology, No. 92 West Dazhi Street, Harbin, Heilongjiang 150001, China; Zhengzhou Research Institute, Harbin Institute of Technology, No. 26 Longyuan East 7th Street, Zhengzhou, Henan 450000, China; School of Mathematics, Harbin Institute of Technology, No. 92 West Dazhi Street, Harbin, Heilongjiang 150001, China; Zhengzhou Research Institute, Harbin Institute of Technology, No. 26 Longyuan East 7th Street, Zhengzhou, Henan 450000, China

**Keywords:** single-cell data, batch correction, biological variation

## Abstract

With the continuous application of single-cell data, effectively correcting batch effects and accurately identifying cell types has emerged as a critical challenge in biomedical research. However, existing methods often struggle to disentangle technical effects from genuine biological variation, limiting their performance on heterogeneous datasets. Here, we introduce single-cell Batch Correction Network (scBCN), an integration framework that combines robust inter-batch similar cluster identification with a deep residual neural network to correct batch effects while preserving biological variability. To evaluate the performance of scBCN, we conduct benchmarking experiments on various simulated and real datasets, demonstrating its superiority in both batch correction and biological variation conservation. Furthermore, scBCN shows its applicability in cross-species and cross-omics data integration, underscoring its potential for uncovering and characterizing cell type-specific gene expression patterns.

## Introduction

The rapid advancement of single-cell RNA sequencing (scRNA-seq) technologies has enabled researchers to profile high-throughput gene expression at the single-cell resolution, thereby uncovering cellular heterogeneity within complex tissues. This innovation has facilitated in-depth investigations into novel cell type identification, transcriptional stochasticity, and gene regulatory network inference at an unprecedented granularity [1–3]. In addition, the emergence of single-cell Assay of Transposase Accessible Chromatin sequencing (scATAC-seq) technologies has provided researchers with a powerful tool to investigate chromatin accessibility and gene regulatory element activity across individual cells, further enriching our understanding of cellular states and functions [4, 5]. However, as increasingly large single-cell datasets are generated, particularly those derived from different experimental batches and platforms, batch effects present a significant challenge for data integration [6]. These effects can confound the interpretation of gene expression patterns, obscure valid biological signals, and compromise the accuracy and reliability of downstream analyses. Therefore, effective batch correction is essential for accurately capturing true biological characteristics and ensuring the credibility and consistency of integrative analyses across studies.

Recently, many computational methods have been developed for batch correction of single-cell data. These methods can be broadly categorized into three major classes. The first class includes similar cell-based methods, such as fastMNN [7], Seurat V3 [8], Scanorama [9], Conos [10], and iSMNN [11]. These methods identify mutual nearest neighbors (MNNs) across batches in a reduced-dimensional space, such as principal component analysis (PCA) or canonical correlation analysis space. These MNN pairs are assumed to represent cells in similar states, facilitating their alignment in subsequent integration steps. For example, Conos constructs global neighborhood graphs across all batches that can be directly utilized for downstream analysis. The second class comprises shared cell type-based methods, including Harmony [12], Liger [13], iMAP [14], and scMC [15]. These methods utilize shared cell types as alignment references to correct batch effects by identifying and adjusting these common cell populations. For instance, LIGER applies integrative non-negative matrix factorization to generate low-dimensional matrices, reducing heterogeneity between datasets by separating shared and non-shared factors. The third class includes deep learning-based methods, such as scVI [16], scANVI [17], scGAN [18], BERMUDA [19], and CarDEC [20], which employ deep neural networks, generative adversarial networks, or variational autoencoders (VAEs) to align data across batches by learning the underlying distribution or embedding space of the data. Despite these advances, existing approaches face important limitations. For heterogeneous single-cell data containing batches with unbalanced cell type compositions, methods based on similar cells or shared cell types may connect two different cell types. Additionally, deep learning-based approaches often suffer from reduced interpretability or strong model assumptions. A recent benchmarking study [21] also highlighted the limitations of these methods in complex integration tasks, underscoring the need for improved strategies that balance batch correction and biological variation conservation.

Here, we propose scBCN (single-cell Batch Correction Network), a deep learning framework for integrating single-cell datasets from multiple heterogeneous batches. scBCN employs a two-stage clustering strategy to accurately and robustly connect similar cell states across heterogeneous batches. Specifically, MNN pairs are identified between batches and extended using a random walk-based approach, which enhances the connectivity among biologically related cells. Based on these extended MNN relationships, scBCN constructs a cluster-level similarity graph using pre-defined high-resolution cell clusters. The graph is then used to connect clusters using spectral clustering. Then, scBCN builds a residual neural network to correct batch effects and embed cells into a unified low-dimensional space. The network leverages the similarity structure derived from the cluster graph to guide training, and applies a Tuplet Margin Loss to enforce intra-cluster compactness and inter-cluster separation. This loss function encourages cells from similar clusters to be embedded closer together while distancing those from dissimilar clusters, ultimately producing a batch-invariant representation that preserves meaningful biological variation. We benchmark scBCN against other state-of-the-art methods using both simulated and real scRNA-seq datasets, demonstrating its superior performance, particularly in biological conservation and batch correction. Additionally, we applied scBCN to several comprehensive analyses of real datasets across different scenarios, highlighting its ability to accurately integrate and identify cell subpopulations in heterogeneous datasets.

## Materials and methods

### Overview of scBCN

**Figure 1:**
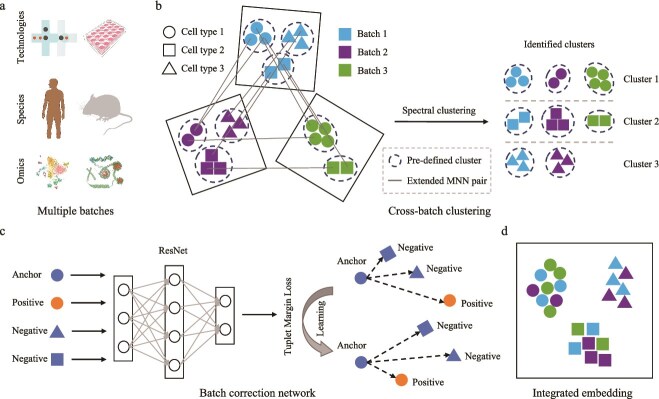
Overview of the scBCN method. (a) scBCN integrates multiple scRNA-seq batches that may come from different technologies, species, or omics. (b) scBCN performs cross-batch clustering to identify similar clusters. (c) scBCN leverages a batch correction network to integrate data, trained using a Tuplet Margin Loss. (d) The integrated embedding by scBCN serves as the input of the downstream analysis.

scBCN is a deep learning-based framework designed to integrate multiple heterogeneous single-cell datasets originating from different experimental batches, technology platforms, species, or omics modalities (Fig. 1a). The framework begins by performing cross-batch clustering, which identifies similar clusters across distinct batches (Fig. 1b). Specifically, scBCN extends MNN pairs across batches through a random walk approach, thereby enhancing inter-batch connections. These extended MNN relationships are used to construct a cluster-level similarity matrix based on the high-resolution cell clusters identified by the Leiden algorithm. Subsequently, spectral clustering is applied to this similarity graph to connect clusters across batches. Then, scBCN builds a batch correction network consisting of a residual neural network (Fig. 1c and Supplementary Fig. S1a). The network is trained using a Tuplet Margin Loss, which imposes a metric learning constraint that pulls cells with the same cluster label closer in the learned embedding space, while simultaneously pushing cells with different labels farther apart. This network yields a batch-corrected low-dimensional representation that preserves biologically meaningful variation and is suitable for a wide range of downstream analyses (Fig. 1d). A detailed illustration of the overall scBCN workflow is also provided in Supplementary Fig. S1b.

### Data preprocessing

The pre-processing for single-cell data closely follows the standard workflow outlined in the Scanpy Python package [22] for scRNA-seq analysis. The workflow applied in this study contains the following steps: (i) quality control and filtering, wherein low-quality cells with fewer than ten genes are excluded, and genes expressed in fewer than three cells are removed; (ii) data normalization, involving the normalization of gene expression levels for each cell by the total expression, subsequently scaled by a factor of 10 000, and log-transformed using the log1p function to reduce skewness; (iii) feature selection, where 2000 highly variable genes that exhibit significant variation across cells are identified. The expression levels of these genes are then scaled by z-score transformation to ensure a mean of zero and a variance of one, facilitating downstream analysis; (iv) PCA is employed to reduce dimensionality. Typically, the top 100 principal components (PCs) are retained, based on empirical evidence, and the resulting low-dimensional data is utilized for cell clustering.

### Cross-batch cell clustering

To align similar cell clusters across different batches and identify rare or novel cell types, scBCN employs a two-stage clustering strategy. In the first stage, for each batch, scBCN performs initial cell clustering using the Leiden algorithm [23], applied to the shared nearest neighbor graph constructed in the PCA-embedded space. The resolution parameter of the Leiden algorithm is crucial for determining the clustering granularity: higher resolution values yield a greater number of finer-grained clusters, thereby enhancing the ability to capture rare but biologically relevant subpopulations. Thus, scBCN sets the resolution to 3.0 to promote the detection of subtle heterogeneity and to uncover potentially meaningful cell states or transitions.

In the second stage, scBCN identifies similar cells across all batch pairs and constructs a cluster-level similarity graph to aggregate the clustering structure obtained in the previous step. To achieve this, scBCN leverages the first 10 PCs to compute pairwise cosine distance between cells, enabling a unified search for MNN pairs across batches. Let $\mathbf{X}_{\text{emb}}=\left (\mathbf{X}^{1},\cdots ,\mathbf{X}^{\mathrm{M}}\right )^{T}$ denote the concatenated matrix of all cells projected into the PCA space, where $\mathbf{X}^{m}$ corresponds to the $m$-th batch, $M$ is the number of batches. For any two batches $a$ and $b$ ($a \leq b$, $a,b\in \{1,\cdots ,M\}$), let $S_{a,b}$ represent the set of MNN pairs between batch $a$ and batch $b$. A pair of cells $(i,j)$, where cells $i$ and $j$ are in batches $a$ and $b$, respectively, forms an MNN pair if and only if 


(1) \begin{align*}&j\in\mathrm{NN}_{k}\left(i,b\right)\wedge i\in\mathrm{NN}_{k}\left(j,a\right),\end{align*}

where $\mathrm{NN}_{k}(i,b)$ represents the set of $k$ nearest neighbors ($k=10$ by default) in batch $b$ of cell $i$. MNN is an effective approach for identifying similar cells across two batches. However, it is sensitive to the selection of the parameter $k$ and may miss correspondences involving rare cell types. To address this limitation, scBCN incorporates a random walk-based expansion of MNN pairs. Let $S_{a,b}^{(0)}$ denote the initial MNN pairs set for batches, and $N(i)=\left \{j|(i,j)\in S_{a,b}^{(0)}\right \}$ represent the MNNs of cell $i$ in batch $a$. A random walk is then performed for $T$ steps ($T=10$ by default), identifying the nearest neighbors of $i$ and $j$ as the new MNNs and generating the new MNN pairs $S_{a,b}^{(t)}$, $t=1,\cdots ,T$. The final set of MNN pairs is: 


(2) \begin{align*}&S_{a,b}=\bigcup_{t=0}^{T}S_{a,b}^{(t)}.\end{align*}

This strategy expands the connectivity landscape between cells across batches, uncovering additional biologically plausible relationships that may be obscured due to technical noise or sampling sparsity. It enhances the robustness and completeness of cell alignment, particularly in the context of rare or sparsely represented populations.

After identifying MNN pairs, scBCN constructs a cluster-level similarity graph, where nodes represent initially identified cell clusters. The edge weight between any two nodes is proportional to the number of MNN pairs linking the two clusters, normalized by their respective cluster sizes. This graph captures both intra- and inter-batch relationships, enabling scBCN to apply spectral clustering [24] to partition the cell clusters. Spectral clustering operates on the eigenstructure of the graph Laplacian, projecting clusters into a low-dimensional space that captures nonlinear data geometry and hierarchical population structure. By leveraging this approach, scBCN effectively integrates cell clusters across heterogeneous batches, reduces batch effects, and preserves meaningful biological variation. The resulting fine-grained cluster assignments provide a strong foundation for subsequent stages of deep batch correction.

### Batch correction network construction

To generate a unified, batch-invariant low-dimensional embedding and achieve accurate cross-batch alignment, scBCN finally constructs a deep batch correction network guided by the global cell clustering structure obtained from the previous spectral clustering step. The architecture comprises two stacked residual blocks, each consisting of five layers: two fully connected layers, two batch normalization layers, and one parametric rectified linear unit (PReLU) activation layer. The residual design allows the network to learn complex relationships while mitigating the risk of vanishing gradients, thereby ensuring stable convergence and efficient training even in high-dimensional settings.

The clustering results obtained from spectral clustering are used as input to train the network. The Tuplet Margin Loss function [25], designed for embedding optimization, guides the training process. This function minimizes the distance between cells with the same label while maximizing the separation between cells with different labels, thereby creating a well-structured embedding space. The Tuplet Margin Loss is defined as follows: 


(3) \begin{align*}&L_{\text{tuplet}}=\log\left(1+\sum_{i=1}^{k-1}\exp{\{d\left(x_{a},x_{p}\right)-d\left(x_{a},x_{n_{i}}\right)\}}\right).\end{align*}

where $d$ is the Euclidean distance between embedding vectors, $x_{a}$ is an anchor cell, $x_{p}$ is a positive sample from the same cluster, and $x_{n_{i}}$ represents negative samples from different clusters.

The training uses a mini-batch stochastic gradient descent approach, further boosting computational efficiency and scalability. Through iterative optimization of the embedding space, the batch correction network effectively aligns cells across different batches while conserving their biological characteristics. The resulting low-dimensional batch-corrected representation supports downstream analyses, such as visualization, cell type annotation, and differential expression analysis.

### Evaluation metrics

To benchmark scBCN against other integrated tools, we employ five commonly used metrics to assess the performance of biological variation conservation [Adjusted Rand Index (ARI) [26], Normalized Mutual Information (NMI) [27], and Average Silhouette Width for cell type (ASW_celltype) [6] and batch effect removal [Local Inverse Simpson’s Index of Batch (iLISI) [12] and Kullback–Leibler divergence of batch mixing (BatchKL) [28]. These metrics are detailed as follows.

ARI quantifies the agreement between the predicted clustering and the ground truth, adjusting for chance alignment. Given a contingency table where $n_{ij}$ denotes the number of cells shared between predicted cluster $i$ and true cell label $j$, the ARI is defined as: 


(4) \begin{align*}&\text{ARI} = \frac{\sum_{ij} \binom{n_{ij}}{2} - \left[ \sum_{i} \binom{a_{i}}{2} \sum_{j} \binom{b_{j}}{2} \right] \big/ \binom{n}{2}}{\frac{1}{2} \left[ \sum_{i} \binom{a_{i}}{2} + \sum_{j} \binom{b_{j}}{2} \right] - \left[ \sum_{i} \binom{a_{i}}{2} + \sum_{j} \binom{b_{j}}{2} \right] \big/ \binom{n}{2}},\end{align*}

where $n$ is the total number of cells, $a_{i} = \sum _{j} n_{ij}$, and $b_{j} = \sum _{i} n_{ij}$. The ARI score ranges from 0 (random clustering) to 1 (perfect clustering match).

NMI measures the mutual dependence between predicted clusters and reference labels. It is defined as: 


(5) \begin{align*}&\text{NMI} = 2 \times \frac{\sum_{ij} \frac{n_{ij}}{n} \log \left( \frac{n \cdot n_{ij}}{a_{i} \cdot b_{j}} \right)}{\sum_{i} \frac{a_{i}}{n} \log \left( \frac{a_{i}}{n} \right) + \sum_{j} \frac{b_{j}}{n} \log \left( \frac{b_{j}}{n} \right)},\end{align*}

where the notations follow those in the ARI definition. NMI values range from 0 (no mutual information) to 1 (perfect agreement).

ASW_celltype quantifies the separation of cells based on their annotated types. For a given cell $i$, the silhouette score is defined as: 


(6) \begin{align*}&s_{i}=\frac{b_{i}-a_{i}}{\max\left\{a_{i},b_{i}\right\}},\end{align*}

where $a_{i}$ is the average distance from cell $i$ to all other cells of the same label, and $b_{i}$ is the minimum average distance between cell $i$ and cells from any other cell type. ASW_celltype is the average $s_{i}$ across all cells and ranges from 0 to 1. Higher values indicate that cells are well-separated by cell type.

iLISI evaluates local batch mixing around each cell. For cell $i$ with cell label $p$, an ideal integration always expects the iLISI to be close to the number of batches the cell label $p$ appears. For batch $b$, we compute the iLISI by 


(7) \begin{align*}&\text{iLISI}_{b}=\frac{1}{\left|C_{b}\right|}\sum_{i\in C_{b}}\left(\left|\text{iLISI}\left(i\right)-N_{i}\right|/N_{i}\right),\end{align*}

where $C_{b}$ denotes the set of cells in batch $b$, $N_{i}$ is the number of batches that the label of cell $i$ appears, and $\left |\text{iLISI}\left (i\right )-N_{i}\right |/N_{i}$ measures the deviation between the batch mixing and the ideal mixing around the cell $i\in C_{b}$.The final iLISI scores are averaged: 


(8) \begin{align*}&\text{iLISI}=\frac{1}{\left|B\right|}\sum_{b\in B}\text{iLISI}_{b}.\end{align*}

KL divergence is used to measure batch mixing based on the embedding space. For $B$ batches, the BatchKL is calculated as: 


(9) \begin{align*}&\text{BatchKL}=\sum_{b=1}^{B}p_{b}\log\frac{p_{b}}{q_{b}},\end{align*}

where $q_{b}$ is the proportion of cells from batch $b$ among all cells, and $p_{b}$ is the local batch proportion inferred from the neighborhood distribution in the embedding. Lower BatchKL values indicate better mixing. For comparability, we normalize BatchKL to the range $[0,1]$.

To summarize performance, we define two composite metrics. We use Biological_conservation to evaluate the preservation of cell-type structure, computed as the average of ARI, NMI, and ASW_celltype: 


(10) \begin{align*}& \text{Biological}{\_}\text{conservation} = \frac{\text{ARI} + \text{NMI} + \text{ASW}{\_}\text{celltype}}{3}\end{align*}

On the other hand, Batch_mixing is the average of iLISI and BatchKL: 


(11) \begin{align*}&\text{Batch}{\_}\text{mixing}=\left(\left(\frac{3-\text{BatchKL}}{3}\right)+\left(\frac{\text{iLISI}}{3}\right)\right)/2\end{align*}

This formulation balances the complementary perspectives of iLISI and BatchKL, accounting for potential metric biases.

## Results

### Simulation study

First, we evaluated the performance of scBCN using two simulated single-cell datasets, including dataset 1 generated using the Splatter R package [29] and dataset 2 used in a comprehensive benchmarking study [21]. We benchmarked our method with eight state-of-the-art methods: fastMNN [7], Harmony [12], Liger [13], Scanorama [9], scANVI [17], scMC [15], scVI [16], and Seurat V4 [30] (see Supplementary Table S1 for details). We applied the uniform manifold approximation and projection (UMAP) algorithm [31] to visualize the integrated data. To quantitatively compare the integration results, we employed three metrics (ARI, NMI, and ASW_celltype) to evaluate the biological variation conservation and two metrics (iLISI and BatchKL) to evaluate the batch mixing (see Materials and methods).

**Figure 2:**
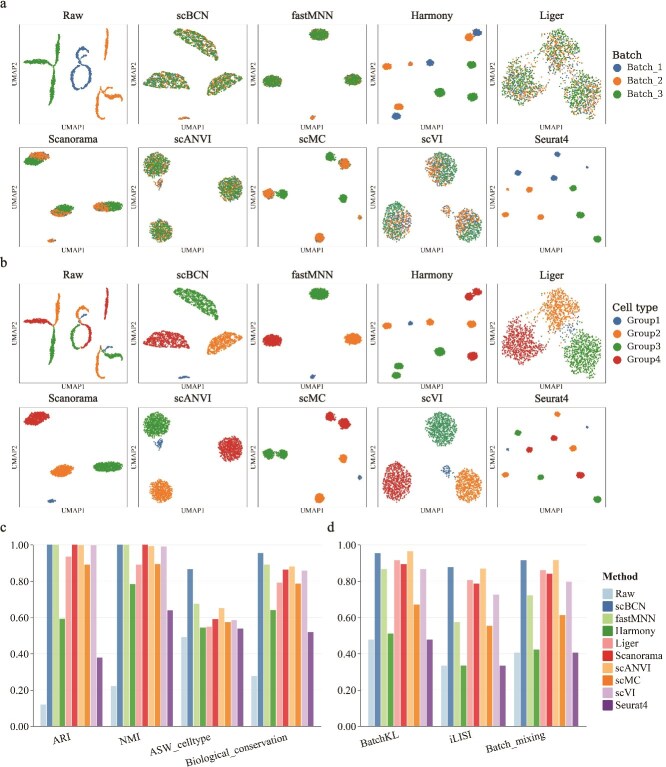
Benchmarking scBCN against other methods using simulated dataset 1. (a) UMAP embeddings of the integrated results of scBCN, fastMNN, Harmony, Liger, Scanorama, scANVI, scMC, scVI, and Seurat V4, with cells colored by batch. (b) UMAP embeddings of the integrated results of scBCN, fastMNN, Harmony, Liger, Scanorama, scANVI, scMC, scVI, and Seurat V4, with cells colored by cell type. (c) The bar plot showing the scores of ARI, NMI, and ASW_celltype for different methods. A higher bar means better performance of biological variation conservation. (d) The bar plot showing the scores of BatchKL and iLISI for different methods. A higher bar means better performance of batch mixing.

In simulated dataset 1, which consists of four cell types and three batches, the raw data exhibited batch effects, with the same cell types showing distinct separation across different batches (Fig. 2a and b). fastMNN, Scanorama, scANVI, scVI, and scBCN successfully integrated the data from these three batches while effectively conserving the boundaries between different cell types. In contrast, other methods either incorrectly connected different cell types or failed to mix batches. Notably, scBCN achieved perfect scores for both ARI and NMI (up to 1.0), and also outperformed all other methods on ASW_celltype and iLISI metrics, demonstrating superior performance in both biological variation conservation and batch correction (Fig. 2c, d and Supplementary Table S2).

For simulated dataset 2, consisting of four cell types and four batches, UMAP visualizations of the raw data revealed substantial batch-induced separation (Supplementary Fig. S2a and b). Among the evaluated methods, only scBCN and scANVI achieved effective mixing across batches. Importantly, scBCN attained the highest values for ARI, NMI, and ASW_celltype, indicating the most accurate preservation of biological variation (Supplementary Fig. S2c and Table S3). While scANVI performed slightly better on batch mixing metrics (Supplementary Fig. S2d), its UMAP embeddings failed to recover the correct cell type structure (Supplementary Fig. S2b), undermining its interpretability. Together, these results demonstrate that scBCN consistently outperforms competing methods on simulated data, offering a strong balance between effective batch correction and faithful preservation of biological signals.

### Benchmark on real datasets

To benchmark scBCN on real datasets, we next evaluated its performance against other methods using two scRNA-seq datasets with known cell type labels and relatively small to moderate data sizes. Specifically, we selected: (i) a mammary epithelial cell dataset composed of three independent studies [32–34], and (ii) a human pancreas scRNA-seq dataset [14].

**Figure 3:**
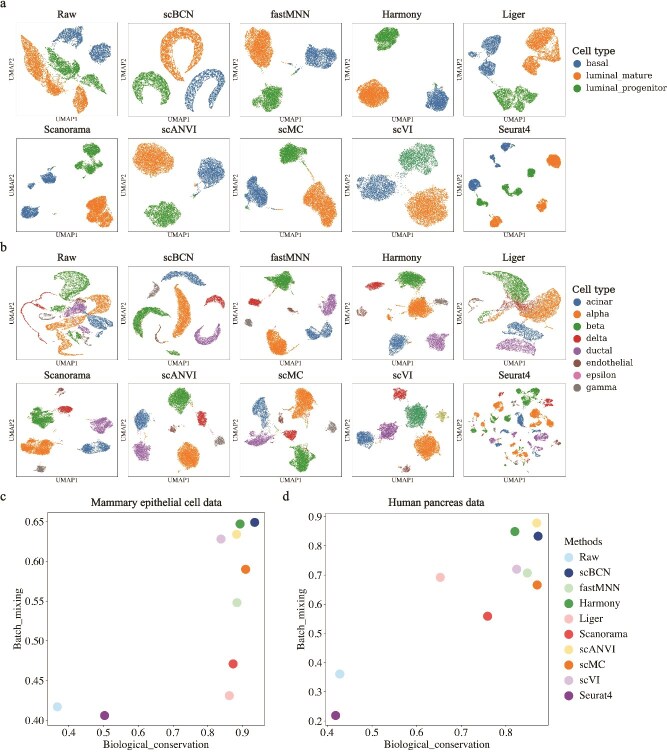
Benchmarking scBCN against other methods using real datasets. (a) UMAP embeddings of mammary epithelial cell dataset integrated by scBCN, fastMNN, Harmony, Liger, Scanorama, scANVI, scMC, scVI, and Seurat V4, with cells colored by cell type. (b) UMAP embeddings of human pancreas dataset integrated by scBCN, fastMNN, Harmony, Liger, Scanorama, scANVI, scMC, scVI, and Seurat V4, with cells colored by cell type. (c) Comparison of evaluation metrics, including biological variation conservation and batch mixing, of the integrated results on the mammary epithelial cell dataset. (d) Comparison of evaluation metrics, including biological variation conservation and batch mixing, of the integrated results on the human pancreas dataset.

The mammary epithelial dataset includes three batches comprising a total of 9288 cells spanning three major cell types. scBCN successfully integrated the batches while preserving clear cell type separation, as evidenced by the well-defined clustering structure in the UMAP embedding (Fig. 3a and Supplementary Fig. S3a). Quantitatively, scBCN achieved the highest scores for both biological conservation and batch mixing, outperforming all other compared methods (Fig. 3c and Supplementary Table S4). These results demonstrate that scBCN not only corrects batch effects effectively but also preserves biologically meaningful cell type distinctions, leading to improved clustering accuracy for the real data integration.

We further validated the performance of scBCN on a more challenging human pancreas dataset, which contains 14,076 cells from eight batches and includes eight distinct cell types. Due to its pronounced batch effects and cell type diversity, this dataset serves as a stringent test for integration algorithms. scBCN effectively mixed cells from different batches while maintaining clear separation among cell types in the embedding space (Fig. 3b and Supplementary Fig. S3b). In the quantitative evaluation, scBCN achieved the highest biological conservation score and ranked third in batch mixing performance (Fig. 3d and Supplementary Table S5). Although scANVI and Harmony obtained slightly higher batch mixing scores, they failed to resolve certain rare cell types in the UMAP space. These results underscore scBCN’s ability to strike a strong balance between effective batch correction and faithful conservation of biological heterogeneity in real-world datasets.

### Cross-species integration

**Figure 4:**
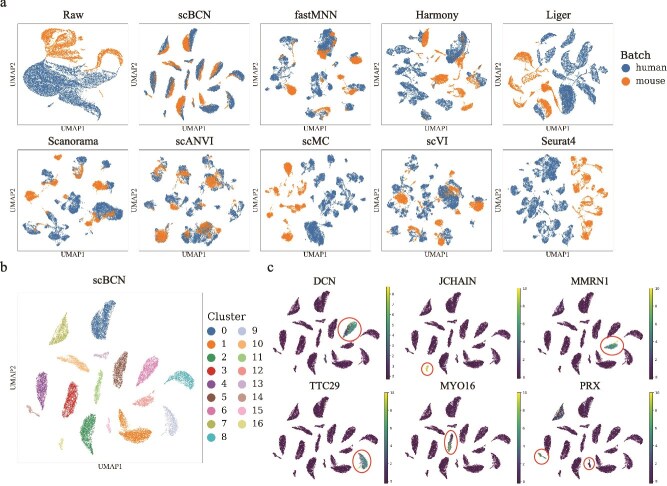
scBCN facilitates cross-species integration of the human and mouse lung datasets. (a) UMAP embeddings of mammary epithelial cell dataset integrated by scBCN, fastMNN, Harmony, Liger, Scanorama, scANVI, scMC, scVI, and Seurat V4, with cells colored by species. (b) UMAP embedding of the scBCN-integrated result, with cells colored by cell cluster. (c) Marker gene feature plot, with points colored by cluster labels derived from scBCN. The plot displays some specific markers for alveolar cells (MYO16), B-cells (JCHAIN), endothelial cells (PRX, MMRN1), and fibroblasts (DCN, TTC29).

Further, we evaluated the ability of scBCN to integrate cross-species single-cell data, a challenging yet biologically informative task. Integrative analysis of such datasets not only enables comprehensive conservation of biological signals across species but also facilitates the discovery of novel or rare cell populations. To this end, we employed two publicly available scRNA-seq datasets derived from lung tissues of humans and mice [35]. After standard preprocessing and dimensionality reduction, we observed that the datasets exhibited minimal overlap in their original feature space, highlighting substantial batch- and species-specific effects. Following integration using scBCN, cells from both species showed considerable alignment in common cell types (Fig. 4a). Moreover, scBCN preserved the delineation of biologically distinct cell populations, effectively capturing cross-species cellular structure (Supplementary Fig. S4a).

In terms of biological variation conservation, scBCN achieved the second-highest ARI and NMI scores, surpassed only by the supervised method scANVI, and ranked first among all unsupervised methods (Supplementary Fig. S4c and Table S6). This demonstrates that scBCN can robustly retain cell-type specificity without relying on label information. Furthermore, scBCN outperformed all competing methods in the ASW_celltype metric, reinforcing its strength in preserving biologically meaningful clustering (Supplementary Figs S4b and S4c). For batch correction, scBCN achieved the highest scores in both BatchKL and iLISI, indicating superior capability in mixing cells across batches while minimizing batch-specific technical artifacts (Supplementary Fig. S4d). These results collectively highlight scBCN’s robust performance in cross-species integration, achieving an effective balance between batch correction and biological conservation.

Importantly, scBCN also demonstrated a unique ability to reveal rare or underrepresented cell subtypes that were missed by other leading methods. For example, in the integrated embedding generated by scBCN, we identified several distinct cell clusters marked by the expression of canonical cell-type–specific genes, including MYO16 (alveolar cells), JCHAIN (B cells), PRX and MMRN1 (endothelial cells), and DCN and TTC29 (fibroblasts) (Fig. 4b and c). The observed expression patterns were consistent with the annotated cell subtypes (Supplementary Fig. S5), supporting the biological relevance of these clusters. Notably, widely used methods such as scANVI and scVI failed to recover the board cell subtypes, further demonstrating that scBCN is capable of integrating complex cross-species transcriptomic features and uncovering fine-grained cellular identities that might be overlooked by other integration frameworks.

### Batch correction across omics

Finally, we evaluated the applicability and generalizability of scBCN in cross-omics data integration, particularly focusing on the joint analysis of scRNA-seq and scATAC-seq data. While scRNA-seq profiles the transcriptional landscape of individual cells, scATAC-seq captures chromatin accessibility, thereby reflecting the regulatory potential of the genome. Integrating these two modalities enables a more comprehensive understanding of cellular states and gene regulation, offering a holistic view of molecular function at the single-cell level.

**Figure 5:**
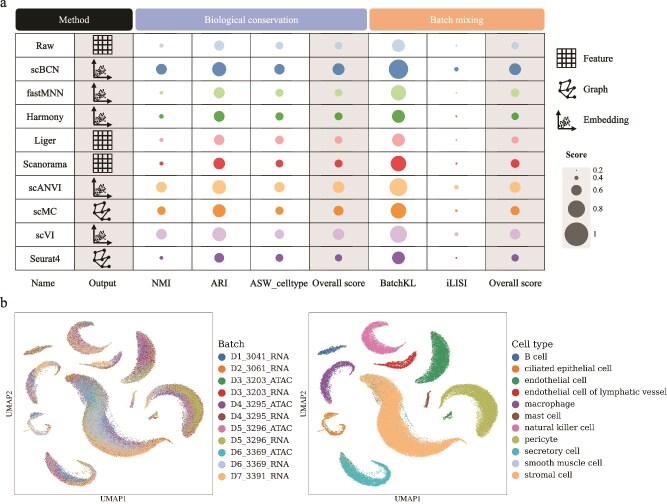
scBCN supports integration of scRNA-seq and scATAC-seq data. (a) Performance comparison of integrated results of the PBMCs data by scBCN and comparative methods, including the output formats of different methods and detailed breakdowns of each metric. Batch correction is represented by the average of two batch-related metrics, while biological conservation is represented by the average of three metrics. (b) UMAP embedding of the scBCN-integrated result, with cells colored by batch (on the left) and cell type (on the right).

To assess the performance of scBCN in this context, we first analyzed a well-characterized dataset of human peripheral blood mononuclear cells (PBMCs), comprising matched scRNA-seq and scATAC-seq data generated using the 10$\times $ Genomics Chromium platform [36]. Visualization of the integrated embedding via UMAP (Supplementary Fig. S6), alongside quantitative evaluation (Fig. 5a and Supplementary Table S7), revealed that scBCN consistently outperformed competing methods in both biological conservation and batch correction. Notably, while scANVI and scVI produced comparable low-dimensional embeddings with relatively high biological conservation scores, their performance still fell slightly short of scBCN. In contrast, other baseline methods showed poor separation of cell types, leading to lower biological conservation scores, and failed to effectively mix batches across omics, resulting in suboptimal batch mixing metrics. These findings underscore the strength of scBCN in preserving cell identity while mitigating modality-specific artifacts, making it particularly well-suited for integrative analyses across omics layers.

To further validate the robustness of scBCN in tissue-specific multi-omics settings, we applied it to matched scRNA-seq and scATAC-seq datasets derived from human post-menopausal fallopian tube (FT) and ovary tissues [37]. These tissues are of particular interest in studies of reproductive aging and related pathologies, where transcriptional programs and chromatin accessibility jointly influence cellular function. After integration with scBCN, the UMAP visualization (Fig. 5b) showed clear delineation of 11 major cell types. Notably, batch effects among technical replicates of the same tissue were effectively removed, while biological variation between tissues was well preserved. These results demonstrate that scBCN not only ensures accurate data harmonization but also retains biologically meaningful signals, thus enabling reliable downstream interpretation.

## Discussion

In this paper, we propose scBCN, an innovative deep learning–based framework that effectively addresses two major challenges in single-cell data integration: batch effect correction and biological signal conservation. The framework is built upon the synergistic integration of two key components: a clustering module and a deep representation learning module.

In the first module, we introduce a two-stage clustering strategy to capture inter-cellular similarity across batches. Notably, we employ a random walk–based extension of MNNs to enhance the robustness and stability of cross-batch cluster identification. This design allows scBCN to better preserve subtle biological signals, particularly those associated with rare or underrepresented cell populations, and significantly improves the resolution of such cell subtypes.

In the second module, we develop a novel batch correction network based on residual neural architectures, which are inherently capable of capturing complex nonlinear relationships while maintaining stability during training. The network is trained using a Tuplet Margin Loss, an advanced metric-learning objective that enhances the discrimination of cell types by pulling together embeddings of similar cells while pushing apart dissimilar ones. This design ensures that the learned embeddings maintain biological fidelity while minimizing batch-induced artifacts. Through the seamless integration of these two modules, scBCN offers a powerful and scalable solution for the integration of diverse single-cell datasets.

scBCN has demonstrated consistently superior performance across a wide range of integration tasks. On both simulated and real datasets, scBCN achieved state-of-the-art performance in terms of biological conservation and batch correction, as measured by multiple quantitative metrics and visualized through UMAP embeddings. Particularly in cross-species integration scenarios, scBCN effectively reconciled the inter-species heterogeneity in gene expression and cell type distribution, successfully integrating human and mouse lung scRNA-seq datasets while uncovering rare and biologically meaningful subpopulations. These results underscore the method’s robustness, adaptability, and generalizability across a variety of data types and biological conditions.

In addition, scBCN shows considerable promise in cross-omics integration, such as the joint analysis of scRNA-seq and scATAC-seq data. By harmonizing transcriptomic and chromatin accessibility profiles, scBCN facilitates a more comprehensive characterization of cellular identity and regulatory state. In both PBMC datasets and tissue-specific datasets from human post-menopausal FT and ovary, scBCN not only effectively removed technical variation across modalities and replicates but also preserved biologically meaningful differences between tissues. These results highlight scBCN’s potential as a versatile tool for integrative multi-omics analysis, particularly in studies aiming to decipher the mechanisms of gene regulatory dynamics.

However, we must acknowledge certain limitations of scBCN. Compared with methods like Seurat v4, scBCN is currently capable of generating only integrated low-dimensional embeddings and cannot provide corrected gene expression data. Consequently, scBCN may not directly support downstream tasks such as differential expression analysis. Future research could further expand the application scope of scBCN by exploring methods to directly eliminate batch effects in single-cell data at the gene expression level, thereby providing a solid foundation for subsequent differential expression analysis and other downstream biological analyses. We anticipate that with continued methodological advancements and integration of new features, scBCN will play an increasingly prominent role in the field of single-cell omics.

## Conclusion

In summary, we propose a novel framework, scBCN, which combines two-layer clustering and deep-learning techniques to effectively remove batch effects in single-cell data analysis. scBCN has been benchmarked on both simulated and real datasets spanning diverse technological platforms, species, and omics modalities. Compared to state-of-the-art integration methods, scBCN demonstrates superior performance in conserving biological variation and correcting batch effects. It consistently outperforms leading tools such as FastMNN, Harmony, Liger, Scanorama, scANVI, scMC, scVI, and Seurat v4. Notably, scBCN not only enables more accurate and efficient integration across batches but also excels in maintaining cell type specificity, even under challenging scenarios such as cross-species data integration, where it successfully identifies rare and subtle cell subtypes that are often missed by other methods. Furthermore, scBCN proves to be highly effective in cross-omics integration, particularly in the joint analysis of scRNA-seq and scATAC-seq data, offering valuable insights into the underlying mechanisms of gene regulation. Given these strengths, we believe that scBCN provides a robust and versatile tool that opens new avenues for future single-cell research.

**Key Points**

- scBCN is a deep learning–based framework for single-cell data integration that adopts a two-stage clustering and batch correction architecture, effectively eliminating batch effects while preserving authentic biological variability.
- scBCN expands mutual nearest neighbor pairs through a random walk strategy to capture broader cellular relationships, thereby enhancing the resolution of rare subpopulations. It employs residual neural networks combined with Tuplet Margin Loss to achieve precise and efficient batch correction with high computational scalability.
- scBCN has demonstrated strong generalizability in both cross-species and cross-modality integration tasks, successfully reconciling gene expression differences between species and retaining biologically meaningful signals across modalities.
- scBCN exhibits robust performance across datasets of varying complexity, maintaining high accuracy and efficiency in diverse experimental settings and across different data scales.

## Supplementary Material

scBCN_Supp_bbaf503

## Data Availability

The scBCN Python package is freely available on GitHub at https://github.com/Jinsl-lab/scBCN. The source code used to reproduce the results presented in this manuscript is also provided there.
