# Supplementary material for: scBCN: deep learning-based batch correction network for integration of heterogeneous single-cell data
Source: Brief Bioinform. 2025 Sep 24;26(5):bbaf503. doi: 10.1093/bib/bbaf503 (PMC12459263; doi:10.1093/bib/bbaf503)
Supplement: scBCN_Supp_bbaf503 [file scbcn_supp_bbaf503.docx]

**Supplementary materials**


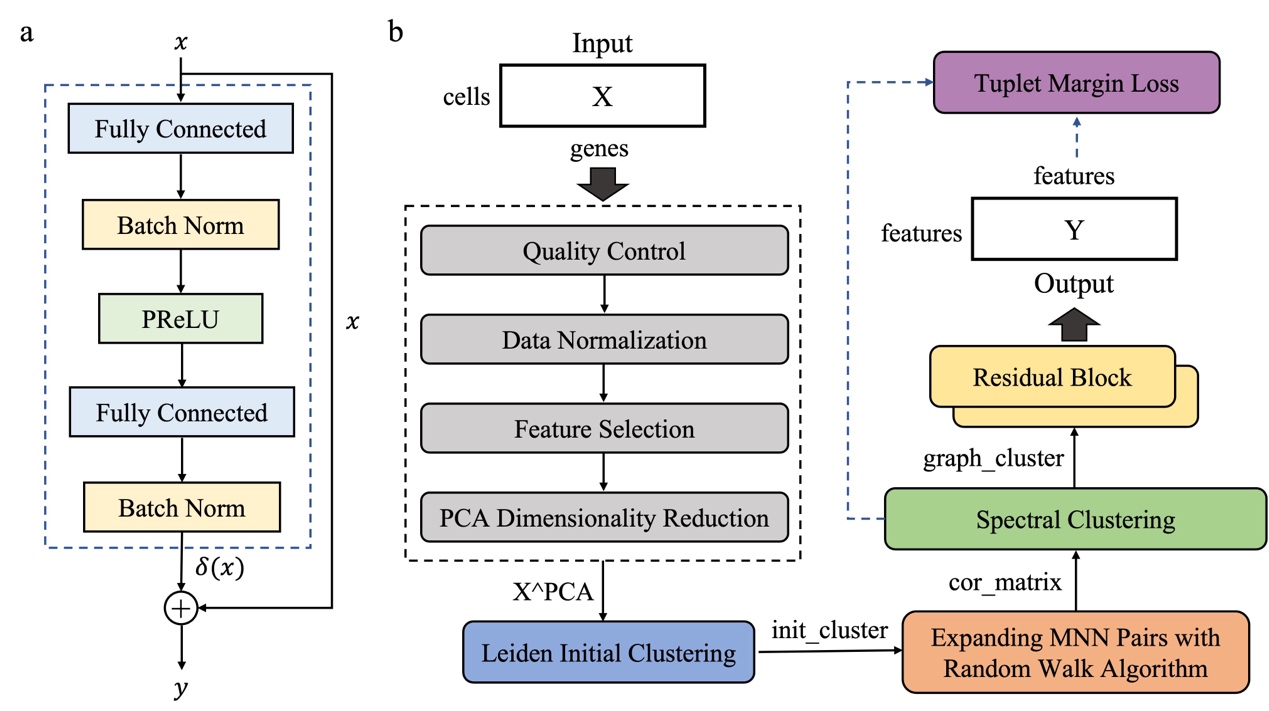


**Fig. S1. The workflow of scBCN.** **a** A residual block consisting of five consecutive layers, including two fully connected layer, two batch normalization layer, and a PReLU activation layer. **b** Schematic diagram of scBCN workflow, including data preprocessing, cross-batch cell clustering and batch correction network.


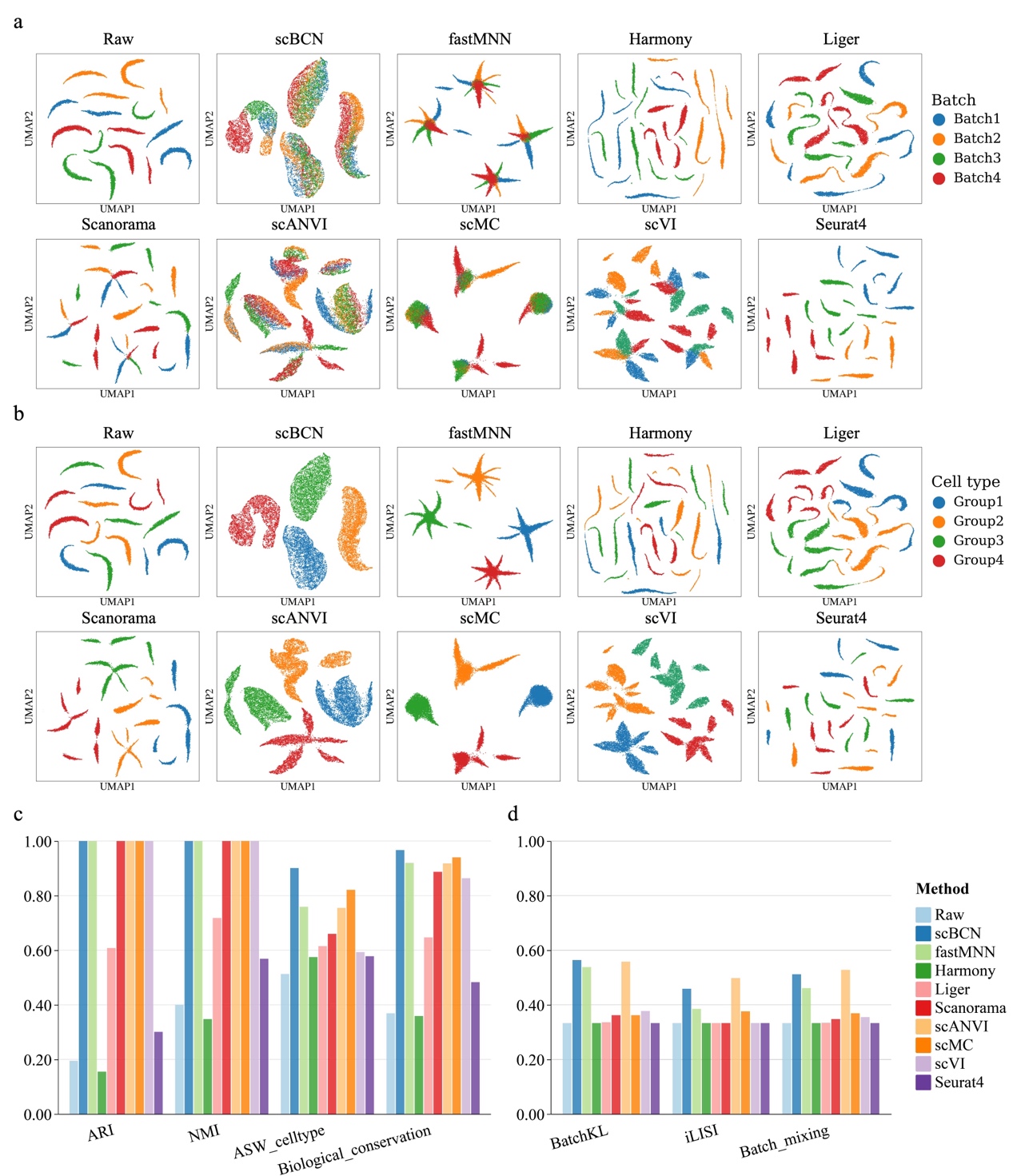


**Fig. S2. Benchmarking scBCN against other methods using simulated dataset 2.** **a** UMAP embedding computed from scBCN, fastMNN, Harmony, Liger, Scanorama, scANVI, scMC, scVI, Seurat V4, in which the points are colored by batch. **b** UMAP embedding computed from scBCN, fastMNN, Harmony, Liger, Scanorama, scANVI, scMC, scVI, Seurat V4, in which the points are colored by cell type. **c** Bar plot shows the score of ARI, NMI and ASW_celltype for different methods. Higher bar means better performance of biology conservation. **d** Bar plot shows the score of BatchKL and iLISI for different methods. Higher bar means better performance of batch mixing.


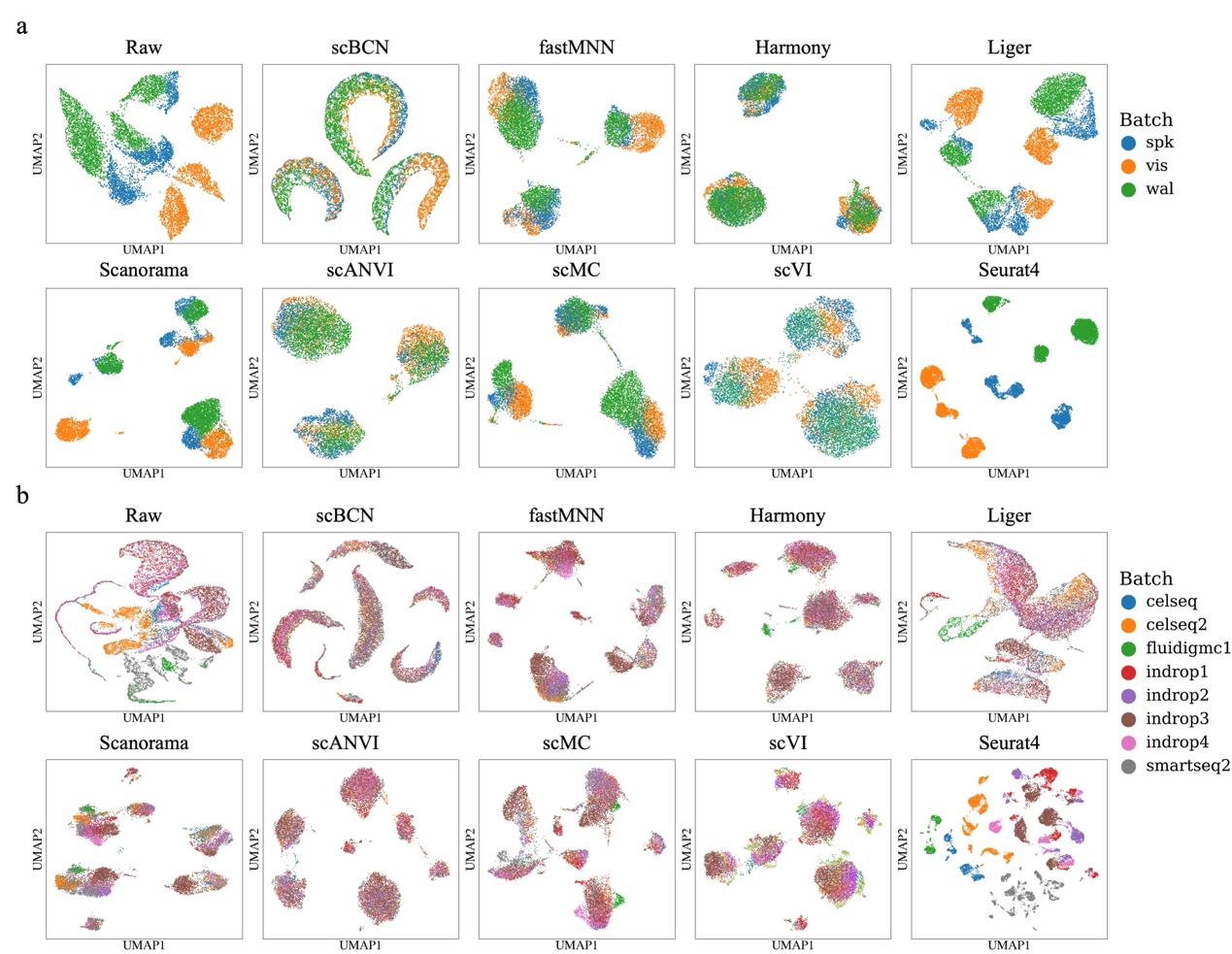


**Fig. S3. Benchmarking scBCN against other methods using real datasets.** **a** UMAP embedding of mammary epithelial cell dataset integration by scBCN, fastMNN, Harmony, Liger, Scanorama, scANVI, scMC, scVI, Seurat V4, in which the points are colored by batch. **b** UMAP embedding of human pancreas dataset integration by scBCN, fastMNN, Harmony, Liger, Scanorama, scANVI, scMC, scVI, Seurat V4, in which the points are colored by batch.


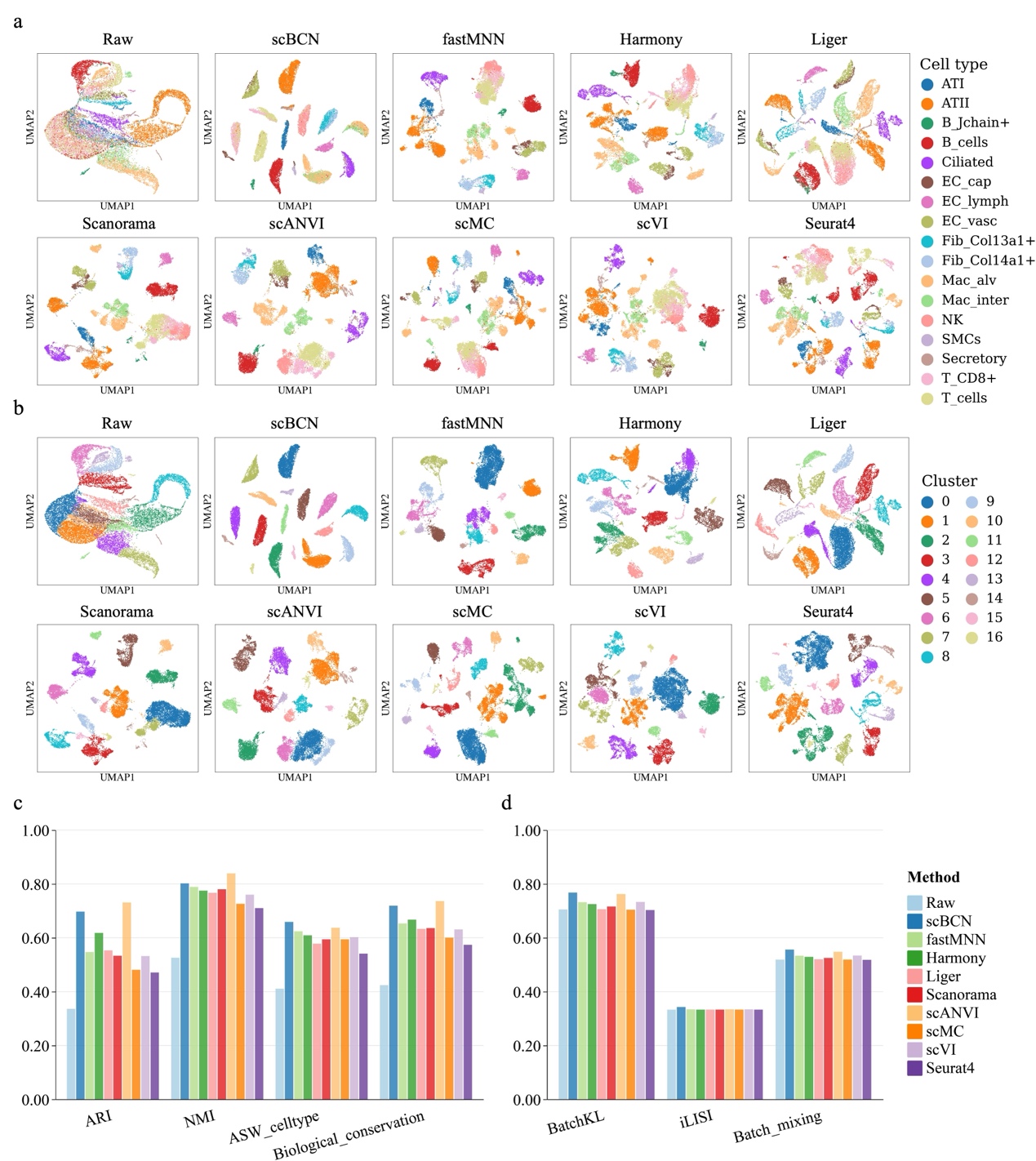


**Fig. S4. scBCN facilitates cross-species integration of the human and mouse lung datasets. a** UMAP embedding of mammary epithelial cell dataset integration by scBCN, fastMNN, Harmony, Liger, Scanorama, scANVI, scMC, scVI, Seurat V4, in which the points are colored by cell type. **b** UMAP embedding of human pancreas dataset integration by scBCN, fastMNN, Harmony, Liger, Scanorama, scANVI, scMC, scVI, Seurat V4, in which the points are colored by cluster label. **c** Bar plot shows the score of ARI, NMI and ASW_celltype for different methods. Higher bar means better performance of biology conservation. **d** Bar plot shows the score of BatchKL and iLISI for different methods. Higher bar means better performance of batch mixing.


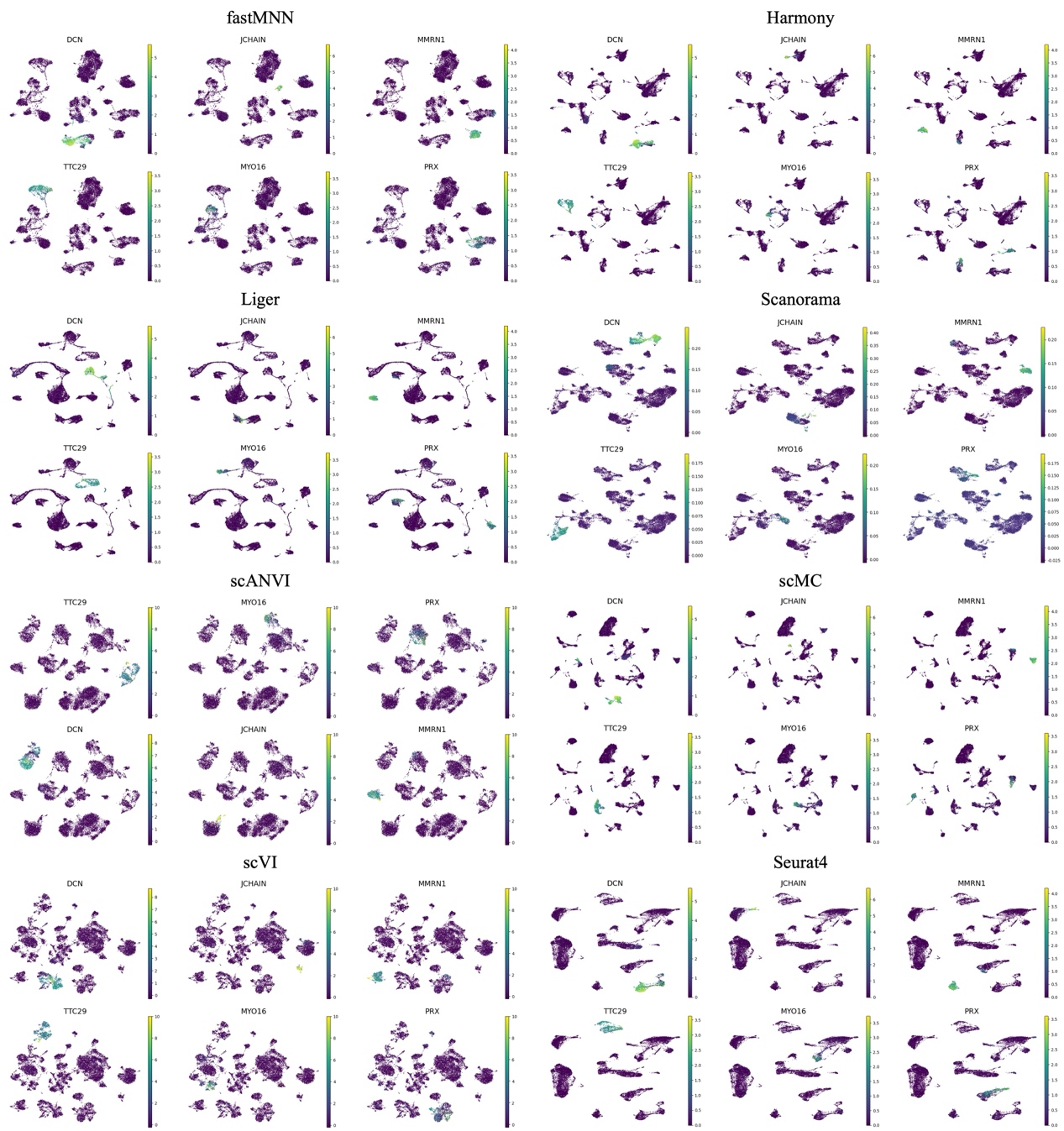


**Fig. S5. Feature plots show some marker genes of different methods for the lung dataset.**


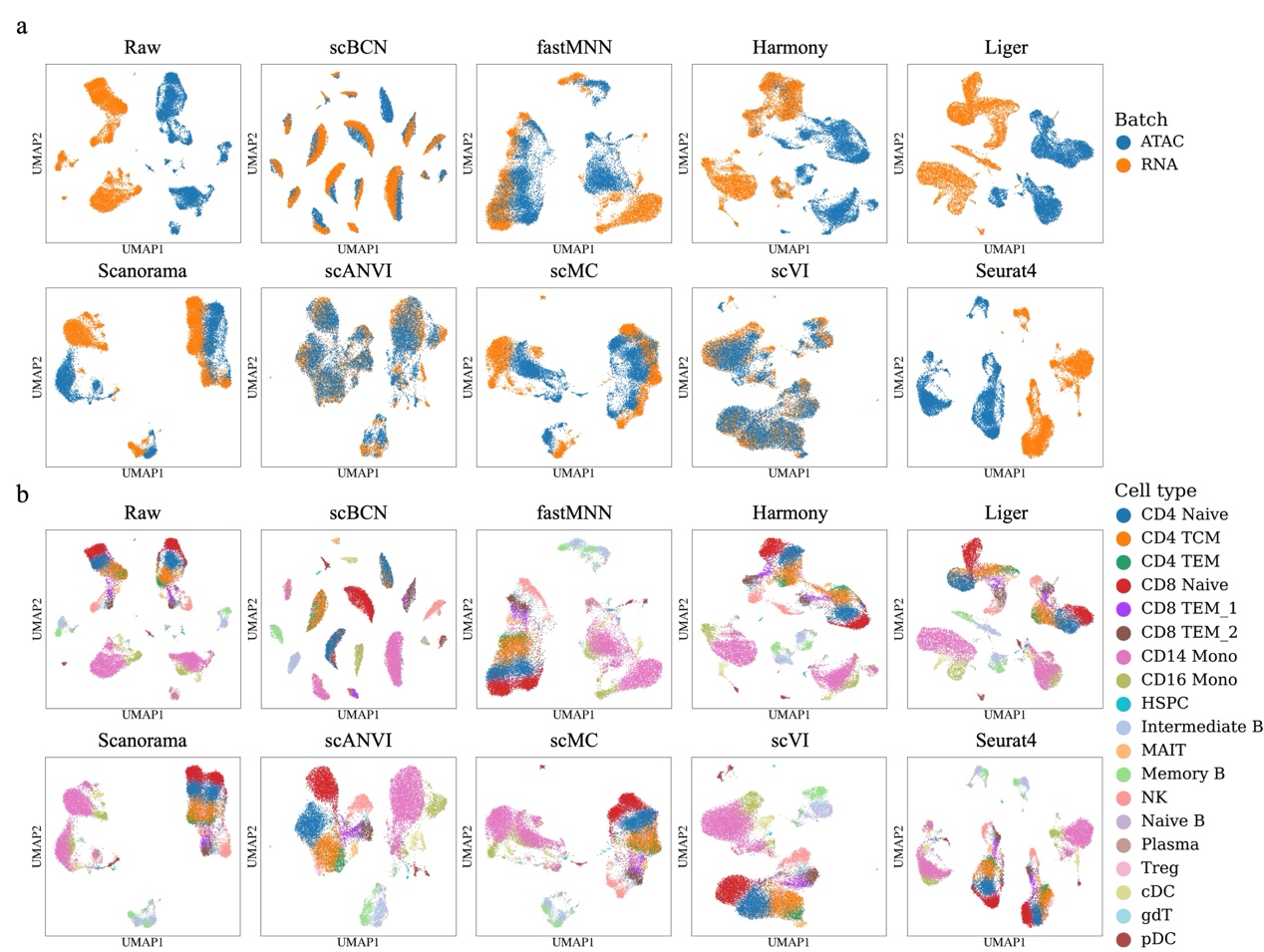


**Fig. S6. scBCN facilitates cross-omics integration of human peripheral blood mononuclear cells (PBMCs) datasets. a** UMAP embedding of mammary epithelial cell dataset integration by scBCN, fastMNN, Harmony, Liger, Scanorama, scANVI, scMC, scVI, Seurat V4, in which the points are colored by batch. **b** UMAP embedding of human pancreas dataset integration by scBCN, fastMNN, Harmony, Liger, Scanorama, scANVI, scMC, scVI, Seurat V4, in which the points are colored by cell type.

**Table S1. Methods compared with scBCN.**

| Method | Version | URL | References |
| --- | --- | --- | --- |
| FastMNN | 1.18.1 | https://bioconductor.org/packages/release/bioc/html/batchelor.html | [1] |
| Harmony | 1.2.0 | https://github.com/immunogenomics/harmony | [2] |
| Liger | 2.1.0 | https://github.com/welch-lab/liger | [3] |
| Scanorama | 1.7.4 | https://github.com/brianhie/scanorama | [4] |
| scANVI | 1.0.4 | https://github.com/scverse/scvitools | [5] |
| scMC | 1.0.0 | https://github.com/amsszlh/scMC | [6] |
| scVI | 1.0.4 | https://github.com/scverse/scvitools | [7] |
| Seurat V4 | 4.4.0 | https://github.com/satijalab/seurat | [8] |

**Table S2. Results of integrating the simulated dataset 1 using scBCN and comparative methods.**

| Method | ARI | NMI | ASW_celltype | BatchKL | iLISI |
| --- | --- | --- | --- | --- | --- |
| Raw | 0.120 | 0.220 | 0.490 | 0.477 | 0.333 |
| scBCN | 1.000 | 1.000 | 0.866 | 0.954 | 0.877 |
| fastMNN | 1.000 | 1.000 | 0.675 | 0.867 | 0.574 |
| Harmony | 0.592 | 0.784 | 0.544 | 0.511 | 0.334 |
| Liger | 0.935 | 0.891 | 0.548 | 0.915 | 0.806 |
| Scanorama | 1.000 | 1.000 | 0.590 | 0.894 | 0.786 |
| scANVI | 0.998 | 0.993 | 0.651 | 0.964 | 0.870 |
| scMC | 0.891 | 0.895 | 0.573 | 0.671 | 0.553 |
| scVI | 0.997 | 0.991 | 0.585 | 0.867 | 0.725 |
| Seurat4 | 0.379 | 0.639 | 0.538 | 0.477 | 0.333 |

**Table S3. Results of integrating the simulated dataset 2 using scBCN and comparative methods.**

| Method | ARI | NMI | ASW_celltype | BatchKL | iLISI |
| --- | --- | --- | --- | --- | --- |
| Raw | 0.195 | 0.400 | 0.513 | 0.333 | 0.333 |
| scBCN | 1.000 | 1.000 | 0.901 | 0.564 | 0.459 |
| fastMNN | 1.000 | 1.000 | 0.759 | 0.538 | 0.385 |
| Harmony | 0.155 | 0.348 | 0.575 | 0.333 | 0.333 |
| Liger | 0.608 | 0.718 | 0.615 | 0.336 | 0.333 |
| Scanorama | 1.000 | 1.000 | 0.660 | 0.362 | 0.333 |
| scANVI | 1.000 | 1.000 | 0.755 | 0.558 | 0.498 |
| scMC | 1.000 | 1.000 | 0.821 | 0.362 | 0.376 |
| scVI | 1.000 | 1.000 | 0.593 | 0.377 | 0.333 |
| Seurat4 | 0.301 | 0.569 | 0.578 | 0.333 | 0.333 |

**Table S4. Results of integrating the mammary epithelial cell dataset using scBCN and comparative methods.**

| Method | ARI | NMI | ASW_celltype | BatchKL | iLISI |
| --- | --- | --- | --- | --- | --- |
| Raw | 0.254 | 0.332 | 0.514 | 0.501 | 0.333 |
| scBCN | 0.985 | 0.968 | 0.853 | 0.751 | 0.547 |
| fastMNN | 0.976 | 0.954 | 0.722 | 0.678 | 0.418 |
| Harmony | 0.989 | 0.976 | 0.715 | 0.752 | 0.541 |
| Liger | 0.989 | 0.974 | 0.623 | 0.527 | 0.334 |
| Scanorama | 0.986 | 0.971 | 0.662 | 0.596 | 0.347 |
| scANVI | 0.976 | 0.953 | 0.721 | 0.725 | 0.542 |
| scMC | 0.983 | 0.965 | 0.779 | 0.712 | 0.467 |
| scVI | 0.976 | 0.951 | 0.587 | 0.732 | 0.523 |
| Seurat4 | 0.404 | 0.449 | 0.657 | 0.479 | 0.333 |

**Table S5. Results of integrating the human pancreas dataset using scBCN and comparative methods.**

| Method | ARI | NMI | ASW_celltype | BatchKL | iLISI |
| --- | --- | --- | --- | --- | --- |
| Raw | 0.440 | 0.579 | 0.264 | 0.389 | 0.333 |
| scBCN | 0.925 | 0.911 | 0.781 | 0.702 | 0.964 |
| fastMNN | 0.955 | 0.932 | 0.656 | 0.637 | 0.777 |
| Harmony | 0.908 | 0.894 | 0.658 | 0.730 | 0.969 |
| Liger | 0.682 | 0.725 | 0.553 | 0.574 | 0.809 |
| Scanorama | 0.800 | 0.874 | 0.604 | 0.482 | 0.636 |
| scANVI | 0.971 | 0.955 | 0.680 | 0.716 | 1.040 |
| scMC | 0.942 | 0.917 | 0.750 | 0.574 | 0.758 |
| scVI | 0.943 | 0.919 | 0.611 | 0.638 | 0.802 |
| Seurat4 | 0.316 | 0.385 | 0.556 | 0.104 | 0.333 |

**Table S6. Results of integrating the human and mouse lung datasets using scBCN and comparative methods.**

| Method | ARI | NMI | ASW_celltype | BatchKL | iLISI |
| --- | --- | --- | --- | --- | --- |
| Raw | 0.336 | 0.526 | 0.411 | 0.705 | 0.333 |
| scBCN | 0.697 | 0.802 | 0.659 | 0.768 | 0.343 |
| fastMNN | 0.547 | 0.789 | 0.624 | 0.732 | 0.334 |
| Harmony | 0.618 | 0.775 | 0.609 | 0.725 | 0.333 |
| Liger | 0.553 | 0.767 | 0.578 | 0.706 | 0.333 |
| Scanorama | 0.533 | 0.780 | 0.594 | 0.716 | 0.333 |
| scANVI | 0.731 | 0.839 | 0.637 | 0.762 | 0.334 |
| scMC | 0.481 | 0.726 | 0.594 | 0.704 | 0.333 |
| scVI | 0.532 | 0.760 | 0.602 | 0.733 | 0.334 |
| Seurat4 | 0.471 | 0.710 | 0.541 | 0.703 | 0.333 |

**Table S7. Results of integrating human peripheral blood mononuclear cells (PBMCs) datasets using scBCN and comparative methods.**

| Method | ARI | NMI | ASW_celltype | BatchKL | iLISI |
| --- | --- | --- | --- | --- | --- |
| Raw | 0.404 | 0.590 | 0.513 | 0.667 | 0.333 |
| scBCN | 0.608 | 0.711 | 0.597 | 0.870 | 0.408 |
| fastMNN | 0.389 | 0.617 | 0.522 | 0.745 | 0.333 |
| Harmony | 0.413 | 0.609 | 0.525 | 0.673 | 0.333 |
| Liger | 0.397 | 0.597 | 0.538 | 0.667 | 0.333 |
| Scanorama | 0.396 | 0.634 | 0.511 | 0.752 | 0.333 |
| scANVI | 0.603 | 0.703 | 0.591 | 0.824 | 0.411 |
| scMC | 0.532 | 0.671 | 0.526 | 0.753 | 0.355 |
| scVI | 0.605 | 0.707 | 0.562 | 0.801 | 0.400 |
| Seurat4 | 0.471 | 0.710 | 0.541 | 0.667 | 0.333 |

**Reference**

1. Haghverdi L, Lun A T L, Morgan M D, et al. Batch effects in single-cell RNA-sequencing data are corrected by matching mutual nearest neighbors. Nature Biotechnology, 2018, 36(5): 421-427.
2. Korsunsky I, Millard N, Fan J, et al. Fast, sensitive and accurate integration of single-cell data with Harmony. Nature Methods, 2019, 16(12): 1289-1296.
3. Welch J D, Kozareva V, Ferreira A, et al. Single-cell multi-omic integration compares and contrasts features of brain cell identity. Cell, 2019, 177(7): 1873-1887. e17.
4. Hie B, Bryson B, Berger B. Efficient integration of heterogeneous single-cell transcriptomes using Scanorama. Nature Biotechnology, 2019, 37(6): 685-691.
5. Xu C, Lopez R, Mehlman E, et al. Probabilistic harmonization and annotation of single‐cell transcriptomics data with deep generative models. Molecular Systems Biology, 2021, 17(1): e9620.
6. Zhang L, Nie Q. scMC learns biological variation through the alignment of multiple single-cell genomics datasets. Genome Biology, 2021, 22(1): 10.
7. Lopez R, Regier J, Cole M B, et al. Deep generative modeling for single-cell transcriptomics. Nature Methods, 2018, 15(12): 1053-1058.
8. Hao Y, Hao S, Andersen-Nissen E, et al. Integrated analysis of multimodal single-cell data. Cell, 2021, 184(13): 3573-3587. e29.
